# Eosinophils Are An Essential Element Of A Type 2 Immune Axis That Controls Thymus Regeneration

**DOI:** 10.1126/sciimmunol.abn3286

**Published:** 2022-03-11

**Authors:** Emilie J. Cosway, Andrea J. White, Sonia M. Parnell, Edina Schweighoffer, Helen E. Jolin, Andrea Bacon, Hans-Reimer Rodewald, Victor Tybulewicz, Andrew N. J. McKenzie, W. E. Jenkinson, Graham Anderson

**Affiliations:** 1 Institute of Immunology and Immunotherapy, University of Birmingham, Birmingham, UK; 2 Francis Crick Institute, London NW1 1AT, UK; 3 MRC Laboratory of Molecular Biology, Cambridge, UK; 4 Division of Cellular Immunology, German Cancer Research Center, 69120 Heidelberg, Germany; 5 Department of Immunology and Inflammation, Imperial College London, London W12 0NN, UK

## Abstract

Therapeutic interventions used for cancer treatment provoke thymus damage and limit the recovery of protective immunity. Here, we show eosinophils are an essential part of an intrathymic type 2 immune network that enables thymus recovery following ablative therapy. Within hours of damage, the thymus undergoes CCR3-dependent colonisation by peripheral eosinophils, which re-establishes the epithelial microenvironments that control thymopoiesis. Eosinophil regulation of thymus regeneration occurs via the concerted action of NKT-cells that trigger CCL11 production via IL4 receptor signalling in thymic stroma, and ILC2 that represent an intrathymic source of IL5, a cytokine that therapeutically boosts thymus regeneration following damage. Collectively, our findings identify an intrathymic network composed of multiple innate immune cells that restores thymus function during re-establishment of the adaptive immune system.

## Introduction

The thymus plays a critical role in the adaptive immune system by generating αβT-cells that respond to invading pathogens and tumours. In the adult thymus, T-cell development is a multi-stage process controlled by cortical and medullary microenvironments (1). For example, the recruitment of lymphoid progenitors to the thymus involves production of CCL19/CCL21, CXCL12 and CCR9, which represent products of thymic stroma (2–4). Following thymus entry, CD4^-^CD8^-^ double negative (DN) progenitors undergo random rearrangements at the T-cell receptor (TCR) β locus, and successful rearrangement enables signalling via the pre-TCR that induces proliferation, *Tcrb* allelic exclusion, *Tcra* gene rearrangement and CD4/CD8 expression (5). Collectively, this generates a large cohort of cortex-resident immature CD4^+^CD8^+^αβTCR^lo^ thymocytes that are screened for their ability to recognise self-MHC via interactions with cortical thymic epithelial cells (cTEC). This process of positive selection produces MHC-restricted CD4^-^ CD8^+^ single positive (SP) and CD4^+^CD8^-^ SP thymocytes that migrate into thymic medullary areas in a CCR7-CCL21 dependent manner (6). Within the thymic medulla, SP thymocytes are screened further for their TCR specificity via interactions with medullary thymic epithelial cells (mTEC) and dendritic cells (DC) which results in negative selection, Foxp3^+^ regulatory T cell (Treg) generation, or continued conventional SP (cSP) thymocyte maturation. Collectively, these events result in the export of self-tolerant, MHC-restricted Recent Thymus Emigrants (RTE), that are incorporated into the naïve peripheral T-cell pool (7).

Importantly, rates of thymus function are not constant throughout life. Instead, intrathymic T-cell production is variable, and reductions in thymus size can occur in multiple settings. For example, age-related thymus atrophy represents a chronic and progressive loss of functional thymic tissue across the lifespan, and this has a major impact on rates of thymic output and new T-cell production in later stages of life (8). In addition, physiological and/or environmental factors including stress, pregnancy, infection and malnutrition can also impact thymus function (9). Interestingly, the response of the thymus to such acute stimuli is reversible, and the transient reduction in thymus size that takes place is followed by a phase of thymus regeneration. Here, cTEC and mTEC thymic microenvironments undergo a phase of recovery, which enables the rate of thymus function to revert to homeostatic levels (10, 11). This balance between thymus damage and regeneration is also important in a number of clinical settings, where therapeutic interventions for disease treatment result in disruption of the immune system. For example, following ablative therapies used alongside bone marrow transplantation for haematological cancer treatment, successful immune reconstitution requires new intrathymic production of a diverse and self-tolerant naïve T-cell pool. Indeed, the sensitivity of thymic microenvironments to damage can limit new T-cell production and create life-threatening secondary immunodeficiencies (12).

Although the thymus possesses regenerative properties to combat these effects (12), the mechanisms that control this process are poorly understood. This limitation has hindered the identification of new cellular and molecular targets to therapeutically improve thymus recovery. Here, we aimed to identify cellular networks in the thymus that control its recovery after damage and examined whether these cell types and/or their products might offer new therapeutic opportunities for thymus recovery. We identify an intrathymic innate cell network consisting of eosinophils, iNKT-cells and ILC2 that is activated following thymus damage, which controls the regeneration of TEC microenvironments to enable the re-establishment of intrathymic T-cell development.

## Results

### Eosinophils Are Essential For Thymus Regeneration

**Figure 1:**
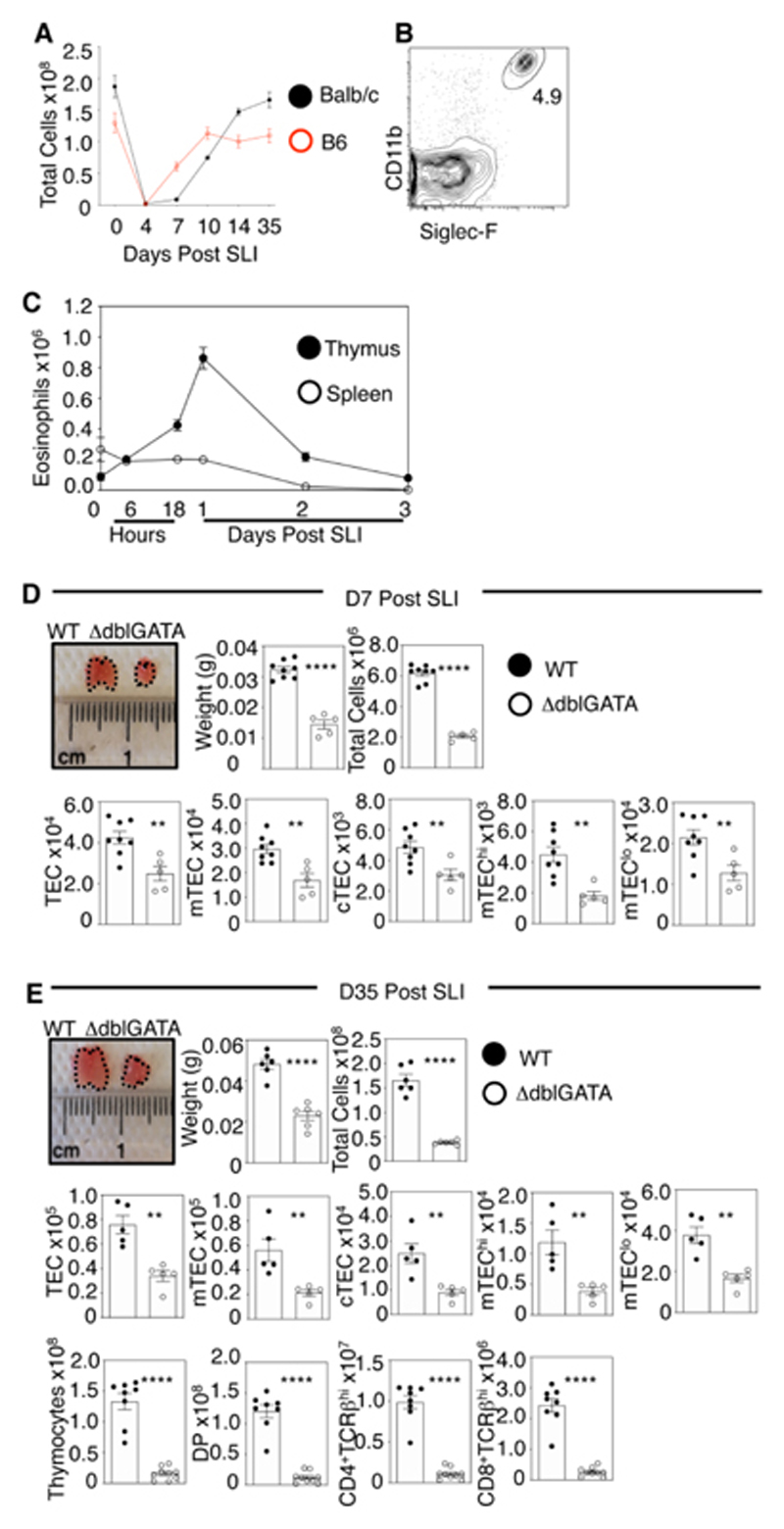
Eosinophil Recruitment to the Thymus is Essential for Thymus Regeneration. (A) Analysis of thymus recovery following sublethal irradiation (SLI) exposure in BALB/c mice and B6 mice where n=6 as a minimum per timepoint across a minimum of two independent experiments. (B) Identification of CD4^-^CD8^-^TCRβ^-^TER119^-^CD11b^+^Siglec-F^+^ eosinophils in steady state WT thymus. (C) Eosinophils in thymus and spleen at d0, 6hr, 18hr, d1, d2, d3 following SLI of BALB/c WT mice, n=6-9 at each time point and data is from three independent experiments. Analysis of thymus recovery (weight, total cellularity, TEC subsets and thymocyte development) in WT and ΔdblGATA mice at d7 (D) and d35 (E) post SLI, n=5-8 and n=6 respectively from 3 independent experiments. All bars show mean ± SEM, ** p<0.01, **** p<0.0001 from an unpaired students t-test.

To study thymus regeneration, we performed total body sublethal irradiation (SLI) of adult wildtype (WT) mice, which represents a well described model with known kinetics of recovery (13, 14). In both BALB/c and B6 mice, we saw a rapid decline in total thymic cellularity, followed by complete recovery by 35 days (d) (Fig. 1A). To identify events that might play a role in the recovery process, we examined cell types present in the thymus 1d post-SLI. Strikingly, we saw a dramatic increase in CD11b^+^Siglec-F^+^ eosinophils (Fig. 1B) in the thymus during the time window following damage (Fig. 1C). This increase was detectable as early as 6 hours, peaked at 1d, and returned to baseline levels by 3d after damage. While numbers of other intrathymic innate immune cells also increased post SLI, the greatest increase was seen with eosinophils (Fig. S1). This surge in thymic eosinophils post-SLI was specific to thymus and not seen in other tissues including spleen (Fig. 1C), lung and small intestine (Fig. S2), suggesting a specialised link between the thymus and eosinophils in the period immediately following irradiation-induced damage. Eosinophils are a subset of granulocytes that possess both destructive pro-inflammatory and regenerative functions (15–17).

To determine whether eosinophils play a role in thymus regeneration, we performed SLI of age- and sex-matched WT and ΔdblGATA mice, the latter lacking eosinophils (including thymic eosinophils, Fig. S3A) due to a deletion within the GATA-1 promoter (18). At steady state, no major differences were noted between WT and ΔdblGATA mice (Fig. S3B). In contrast, analysis at 7d and 35d post-SLI showed thymus regeneration was significantly impaired in ΔdblGATA mice, with reduced thymic size and weight, and reduced numbers of cTEC/mTEC and CD4/CD8 thymocyte subsets including CD4^+^CD8^+^ thymocytes that indicate recovery of thymopoiesis (Fig. 1D, E). As with ΔdblGATA mice, and despite no steady state difference in total thymus cellularity compared to WT, we also saw impaired thymus regeneration in IL-5-deficient mice (Fig. S4), where eosinophil expansion in response to inflammatory stimuli is impaired. Interestingly, while splenic T-cell numbers were comparable in WT and ΔdblGATA at steady state, reduced T-cell development in ΔdblGATA mice following SLI impaired re-establishment of the peripheral αβT-cell pool (Fig. S5). Further work is required to examine whether this impacts immune responses following damage. Failed thymus regeneration in ΔdblGATA mice was evident up to 98d post-SLI, highlighting a sustained impact of eosinophil-deficiency on thymus recovery (Fig. S6). Thus, thymus damage invokes a rapid and transient surge in the intrathymic availability eosinophils, which is essential for long-term recovery of TEC thymic microenvironments that support thymopoiesis.

### Rapid and Transient Eosinophil Recruitment To The Thymus Occurs Following Damage

**Figure 2:**
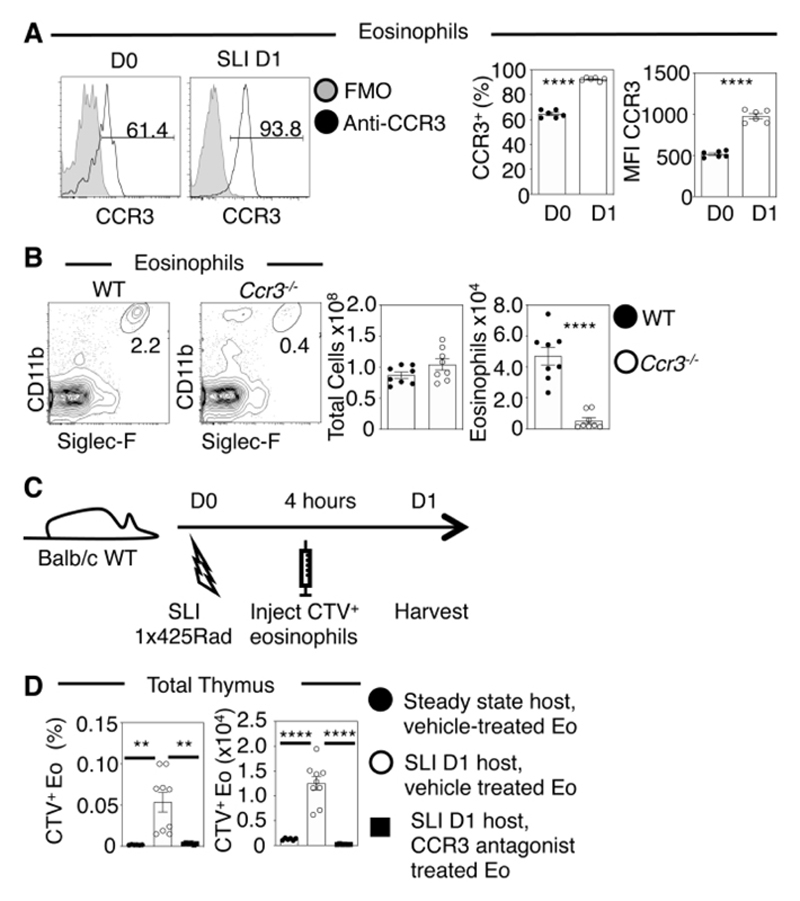
CCR3 Expression by Eosinophils Regulates their Thymus Homing. (A) CCR3 expression by thymic eosinophils at d0 and d1 post SLI, with proportional and MFI analysis, n=6 from three independent experiments. Grey histograms represent control staining levels. (B) Thymic eosinophils in WT and *Ccr3^-/-^* mice, with total cellularity and eosinophil numbers also shown, n=8 from two independent experiments. (C) Experimental setup for transfer of eosinophils generated from bone marrow cultures. Cell Trace Violet (CTV) labelled cells were IV transferred into mice four hours following SLI, and mice were harvested 24 hours after initial SLI. (D) Quantitation of CTV-labelled transferred eosinophils detected in the thymus of steady state mice, and in mice treated with SLI, as indicated with analysis conducted using a one-way ANOVA with Bonferroni post-test. In the latter, eosinophils were pre-incubated in either vehicle alone, or 10mM of the CCR3 antagonist SB328437 prior to injection. Data was obtained from a minimum of four independent experiments where n=6-9. All bars show mean ± SEM, ** p<0.01, **** p<0.0001 from an unpaired students t-test unless otherwise specified.

Given the essential role for eosinophils in thymus regeneration, we next examined the mechanisms determining their intrathymic availability. Increases in the number of thymus eosinophils could result from local expansion or their recruitment from peripheral sites. For eosinophil migration between tissues, CCR3 is an important chemokine receptor that is expressed by eosinophils, which has multiple chemokine ligands, CCL9/CCL10/CCL11, that belong to the Eotaxin subfamily (19). Flow cytometric analysis using an anti-CCR3 antibody showed that CCR3 was detectable on eosinophils in multiple tissues including thymus (Fig. 2A), spleen, lung and small intestine (Fig. S7). At 1d post-SLI, the proportion of CCR3^+^ thymic eosinophils increased, as did their level of CCR3 expression (Fig. 2A). Consistent with a role for CCR3 in their migration, despite comparable total thymus cellularity, eosinophils were almost absent from the thymuses of *Ccr3* ^-/-^ mice (Fig. 2B).

To investigate the role of CCR3 in eosinophil recruitment following damage, we performed cell transfer experiments with Cell Trace Violet (CTV)-labelled eosinophils (Fig. 2C). While few labelled eosinophils entered the thymus of untreated mice, they were readily detectable in the thymus of mice receiving SLI (Fig. 2D). Most significantly, pre-treatment of eosinophils with a CCR3 antagonist (20) prior to transfer into SLI-treated mice completely abrogated recruitment to the thymus (Fig. 2D). Thus, irradiation-induced damage induces rapid recruitment of peripheral eosinophils to the damaged thymus, which occurs in a CCR3-dependent manner. Such findings demonstrate important interplay between the thymus and peripheral tissues in the appearance of transient intrathymic eosinophilia that is essential for thymus regeneration.

### IL4Rα Controls Eosinophil Recruitment During Thymus Damage

**Figure 3:**
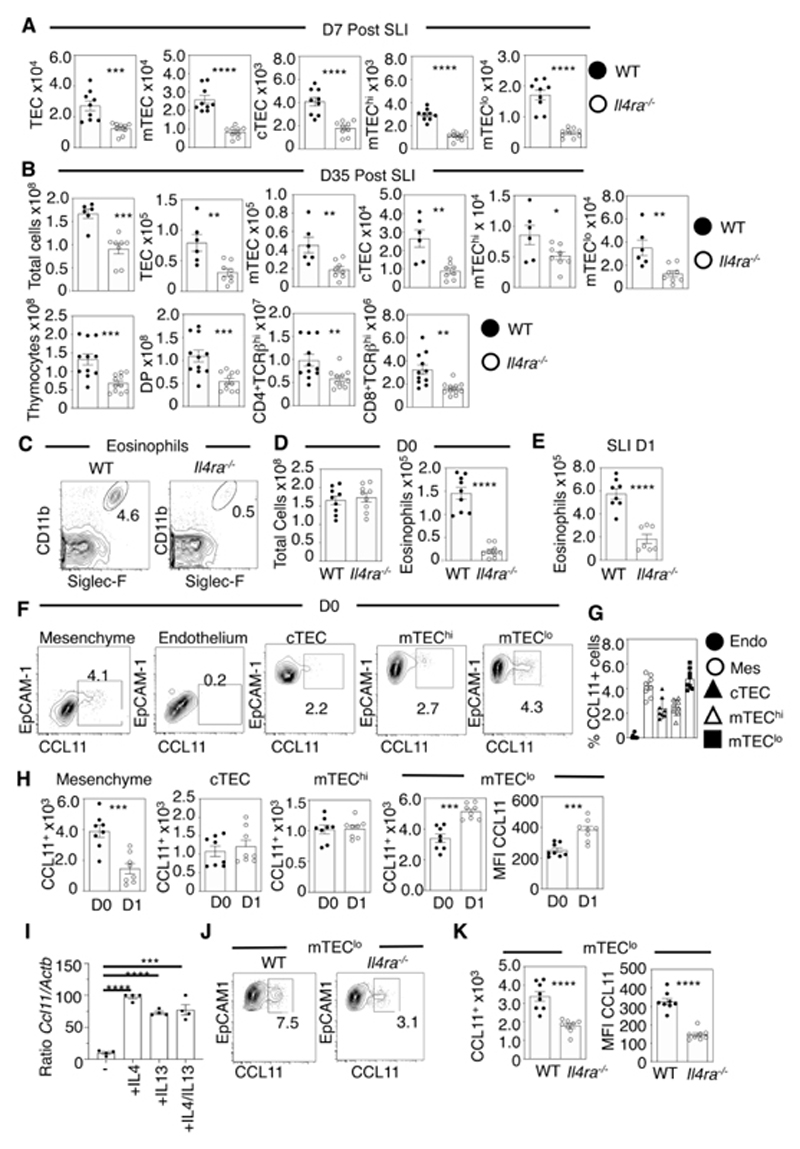
IL4Rα Signalling Stimulates CCL11 Production by mTEC. Analysis of TEC subsets in WT and *Il4ra^-/-^* mice at d7 post SLI (A), n=9, and (B) analysis of TEC (n=6-8) and T-cell development at d35 (n=11). (C) Representative FACS plots of thymic eosinophils in steady state WT and *Il4ra^-/-^* mice. Quantitation of thymus cellularity and thymic eosinophils at d0 (D) and d1 (E) in WT and *Il4ra^-/-^* mice, n=9 at d0, n=7-8 at d1. (F) Representative FACs plots of CCL11 expression in thymic stromal populations, showing proportions (G) and quantitation at d0 and d1 (H), with MFI of CCL11 for mTEC^lo^, n=8. (I) qPCR analysis of *Ccl11* mRNA expression E15 2-deoxyguanosine thymic organ cultures, treated for 4 days +/- IL4/IL13, with analysis conducted using a one-way ANOVA with Bonferroni post-test. (J) Representative FACS plots for expression of CCL11 by mTEC^lo^ in WT (left panel) and *Il4ra^-/-^* (right panel) with quantitation shown in (K), n=8. All data from at least 2-3 independent experiments. All bars show mean ± SEM, * p<0.05, ** p<0.01, *** p<0.001, **** p<0.0001 from an unpaired students t-test unless otherwise specified.

In tissues such as lung, expression of eosinophil chemo-attractants is controlled by the type 2 IL4 Receptor (type 2 IL4R). This cell surface receptor consists of IL4Rα/IL13Rα1 chains and binds the type 2 cytokines IL4/IL13 (21, 22). Previously, we showed that the type 2 IL4R is expressed in the thymus by thymic stroma, including thymic epithelium (23). Hence, we examined whether a type 2 IL4R/CCR3 ligand axis may control thymic eosinophil availability and regeneration. First, we performed SLI on WT and *Il4ra^-/-^* mice, and analysed thymus regeneration as described above. We saw significantly impaired thymus recovery in *Il4ra* ^-/-^ mice at d7 (Fig. 3A). This occurred alongside a sustained reduction in TEC compartments and CD4/CD8 thymocyte subsets at d35 (Fig. 3B). Interestingly, thymic eosinophils were significantly reduced in steady-state *Il4ra* ^-/-^ mice (Fig. 3C, D), and even though they increased in *Il4ra* ^-/-^ mice following SLI (Fig. 3D, E), they remained significantly lower compared to WT, and this increase was not sufficient to induce a normal programme of thymus regeneration (Fig. 3A, B). Thus, IL4Rα may regulate both steady-state and post-damage thymic eosinophil numbers, and diminished thymic eosinophils in *Il4ra* ^-/-^ mice may explain their failure in thymus regeneration.

To investigate whether defective thymus regeneration and the paucity of thymic eosinophils in *Il4ra^-/-^* mice is linked to control of eosinophil chemo-attractants, we examined the relationship between IL4R and intrathymic expression of CCR3 ligands. While murine *Ccl26*(Eotaxin-3) is a pseudogene (24), *Ccl24* (Eotaxin-2) mRNA was undetectable in thymic stroma and thymocytes (Fig. S8). In contrast, flow cytometric analysis detected CCL11 (Eotaxin-1) in multiple thymic stromal subsets at steady state, including cTEC, mTEC^lo^, mTEC^hi^ and EpCAM1^-^ podoplanin^+^ mesenchyme, but not CD31^+^ endothelium (Fig. 3F, G). Thus, CCL11 appears to be the predominant intrathymic CCR3 ligand, which is consistent with the paucity of thymus eosinophils in *Ccl11^-/-^* mice (25), and it is differentially expressed in distinct thymic subsets. While numbers of CCL11^+^ mesenchyme, cTEC and mTEC^hi^ remained the same or were reduced 1d post-SLI (Fig 3H), numbers of CCL11^+^ mTEC^lo^ were increased, together with an increase in CCL11 MFI in these cells (Fig. 3H). To examine CCL11 production in relation to IL4Rα, we first stimulated TEC in 2dGuo-treated FTOC with IL4/IL13, which was sufficient to induce *Ccl11* mRNA expression (Fig. 3I). Moreover, analysis of CCL11^+^ mTEC in steady-state WT and *Il4ra^-/-^* mice showed that CCL11^+^ mTEC^lo^ in *Il4ra^-/-^* mice were significantly decreased, as was their level of CCL11 expression (Fig. 3J, K). Thus, while multiple thymic stromal cell types express CCL11 in the steady state, mTEC^lo^ represent a particular subset that increases in frequency and CCL11 production post-damage in an IL4Rα-dependent manner.

**Figure 4:**
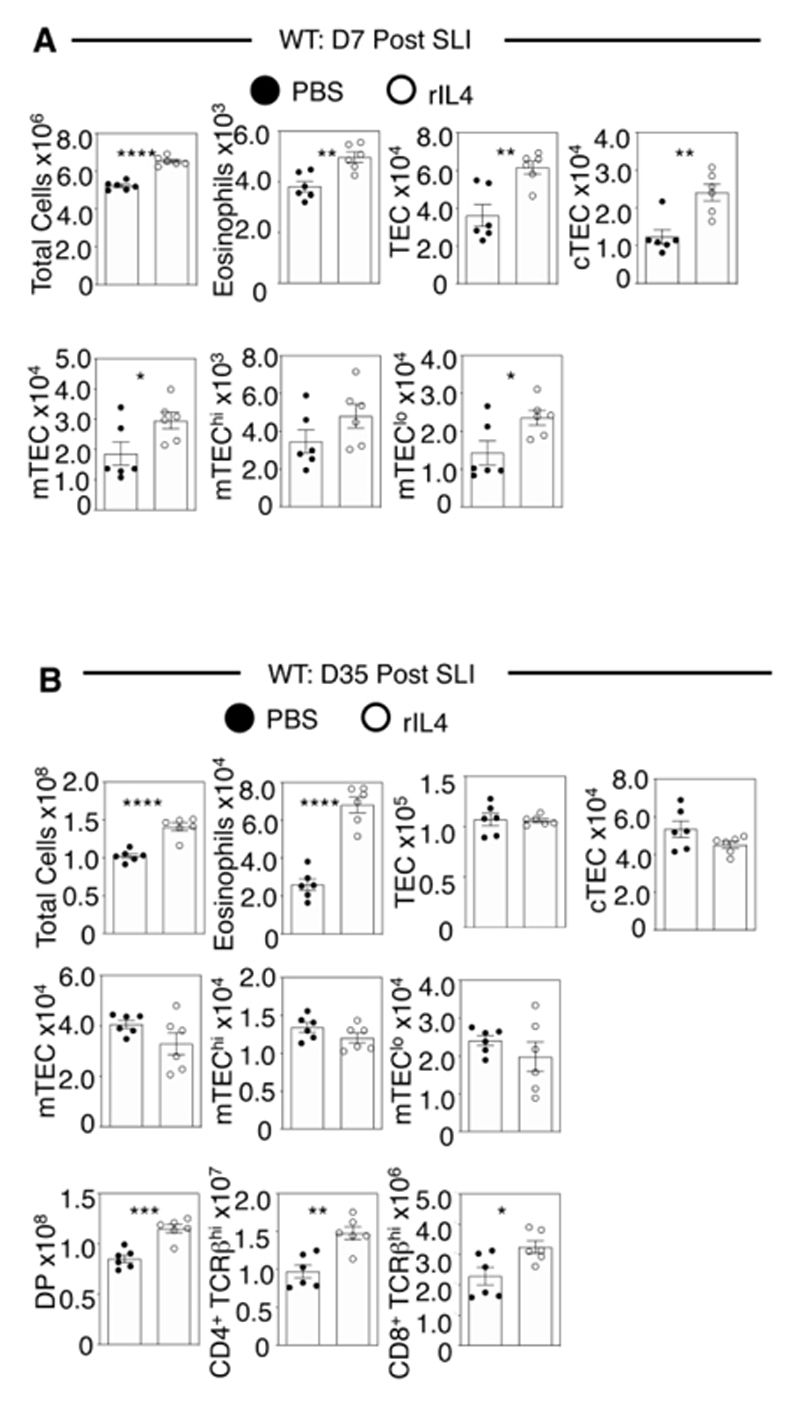
Recombinant IL4 Enhances Thymus Recovery. WT mice were injected with PBS or recombinant IL4 (rIL4), sub-lethally irradiated and harvested d7 (A) or d35 (B) post SLI. Analysis of total thymus cells, eosinophils, TEC and thymocyte subsets was performed, n=6. All data generated was from at least 2-3 independent experiments. All bars show mean ± SEM, * p<0.05, ** p<0.01, *** p<0.001, **** p<0.0001 from an unpaired students t-test.

To further study the importance of IL4/IL4Rα signalling in thymus regeneration, we examined the effects of IL4 administration during thymus regeneration *in vivo.* We injected SLI-treated WT mice with recombinant IL4 using a dosing regimen (Fig. S9) used previously to study IL22 involvement in thymus regeneration (13). Interestingly, IL4 increased thymus cellularity, intrathymic eosinophils and cTEC/mTEC numbers at day 7 post-SLI (Fig. 4A). These effects were also evident at d35 post-SLI and were accompanied by increased thymocyte numbers (Fig. 4B). Collectively, these findings show that IL4Rα plays an important role in thymus regeneration, and IL4 administration enhances this process. Our findings on *Il4ra^-/-^* mice do not allow us to fully discriminate between involvement of the type 1 and/or type 2 IL4R complex, and future experiments analysing thymus regeneration in *Il13ral^-/-^* mice would facilitate this. However, given the expression of IL4Rα/IL13Rα1/IL13Rα2 by thymic stromal cells (23), and their IL4Rα-dependent expression of CCL11 (Fig. 3I), our data suggests the type 2 IL4R regulates CCL11 in thymic stroma, that then controls intrathymic availability of eosinophils required for thymus recovery following damage.

### Thymus Tuft Cells Are Dispensable For Thymus Regeneration

**Figure 5:**
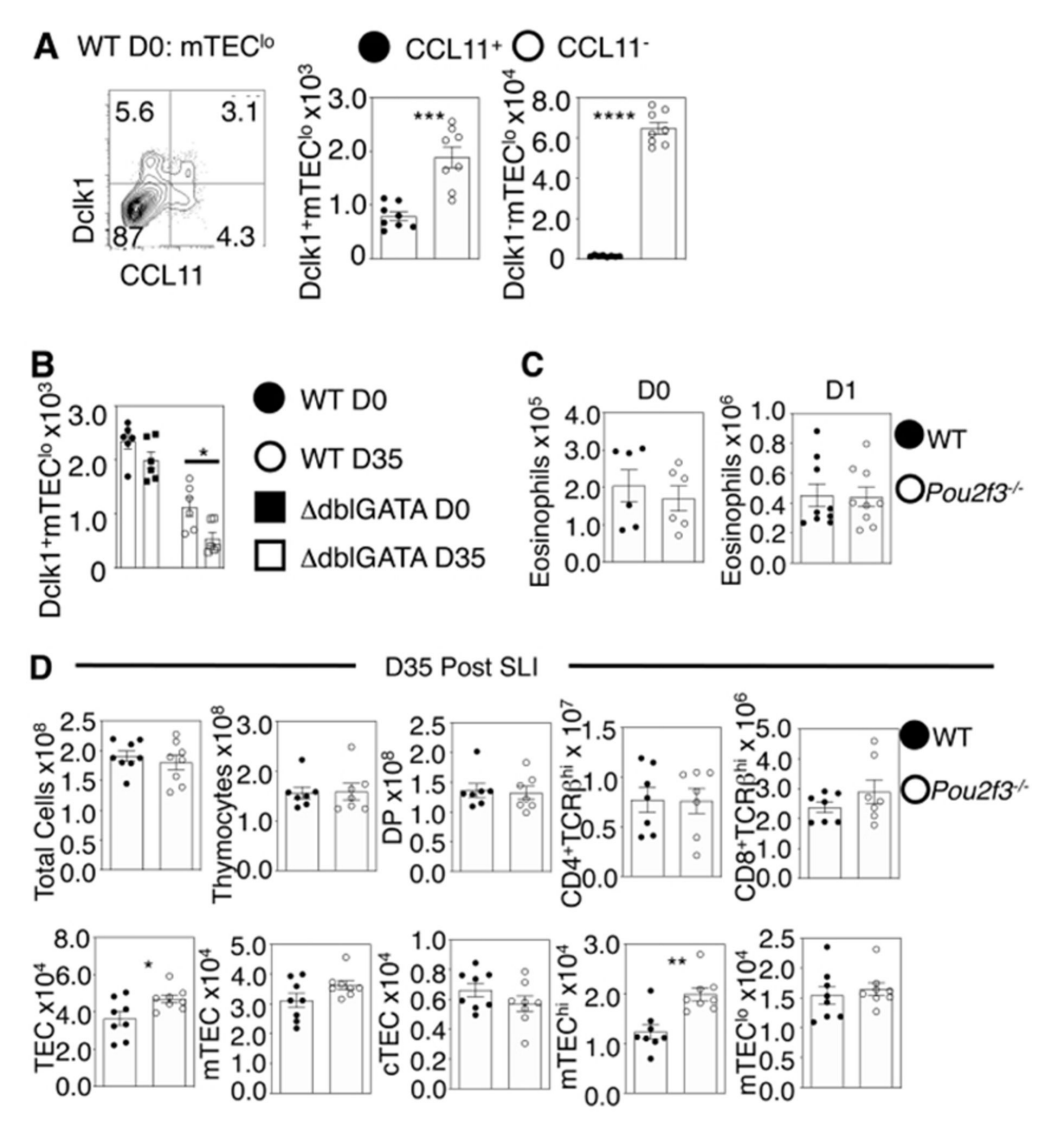
Tuft Cells Are Dispensable For Thymus Regeneration. (A) Analysis and quantitation of CCL11 and Dclk1 expression in mTEC^lo^ of WT mice, n=8 across two independent experiments. (B) Quantitation of Dclk1^+^mTEC^lo^ in WT and Δdb1GATA mice at d0 and d35 post SLI, data from at least two experiments, n=6, analysis was conducted using a one-way ANOVA with Bonferroni post-test. Eosinophil analysis in WT and *Pou2f3^-/-^* mice (C) at d0 and d1 post SLI, n=6 as a minimum across two independent experiments. Analysis of TEC and T-cell development in WT and *Pou2f3^-/-^* mice (D) at d35 post SLI exposure. Analysis was from two independent experiments where n=7-8. All bars show mean ± SEM, * p<0.05, ** p<0.01 from an unpaired students t-test, unless otherwise specified.

Recently, analysis of thymus cell types by RNA sequencing has shown that TEC populations are highly complex and heterogeneous. For example, analysis of the mTEC compartment has revealed the presence of tuft-like cells in the thymus (26, 27). In gut, these cells play a key role in controlling tissue homeostasis and responses to parasitic infection via their involvement in type 2-mediated immunity. In thymus, the functional importance of tuft cells is not fully understood, and while they have been shown to influence both iNKT and ILC2 populations in the steady state, their possible involvement in thymus regeneration has not been addressed (26, 27). As CCL11^+^ cells are contained within the mTEC^lo^ fraction (Fig. 3F) where tuft cells are known to reside (26, 27), we analysed CCL11 alongside the tuft cell marker Dclk1. Interestingly, in the steady state, while the majority of Dclk1^-^ non-tuft mTEC^lo^ were CCL11^-^, Dclk1^+^ tuft cells could be separated into CCL11^+^ and CCL11^-^ subsets (Fig. 5A). Moreover, thymic tuft cell numbers were comparable in steady-state WT and ΔdblGATA mice (Fig. 5B), and thymic eosinophil numbers were also comparable in steady state WT and tuft cell-deficient *Pou2f3^-/-^* mice (Fig. 5C). Thymus eosinophil numbers at d1 post-SLI were also similar in WT and *Pou2f3^-/-^* mice (Fig. 5C), suggesting that increased thymic eosinophil availability in the thymus can occur independently of tuft cells. However, by d35 post-SLI, thymus tuft cells were reduced in both WT and ΔdblGATA mice compared to untreated controls, with a greater deficiency seen in the latter (Fig. 5B). To directly examine the possible involvement of tuft cells in thymus regeneration, we compared post-SLI thymus recovery in WT and *Pou2f3^-/-^* mice. Despite CCL11 production by thymic tuft cells, and their reduction following SLI, thymus regeneration in tuft cell-deficient *Pou2f3^-/-^* mice was comparable to WT (Fig. 5D), suggesting that thymic tuft cells are not essential for thymus recovery after damage.

### iNKT-cells Trigger IL4Rα-Mediated Signalling For Thymus Regeneration

**Figure 6:**
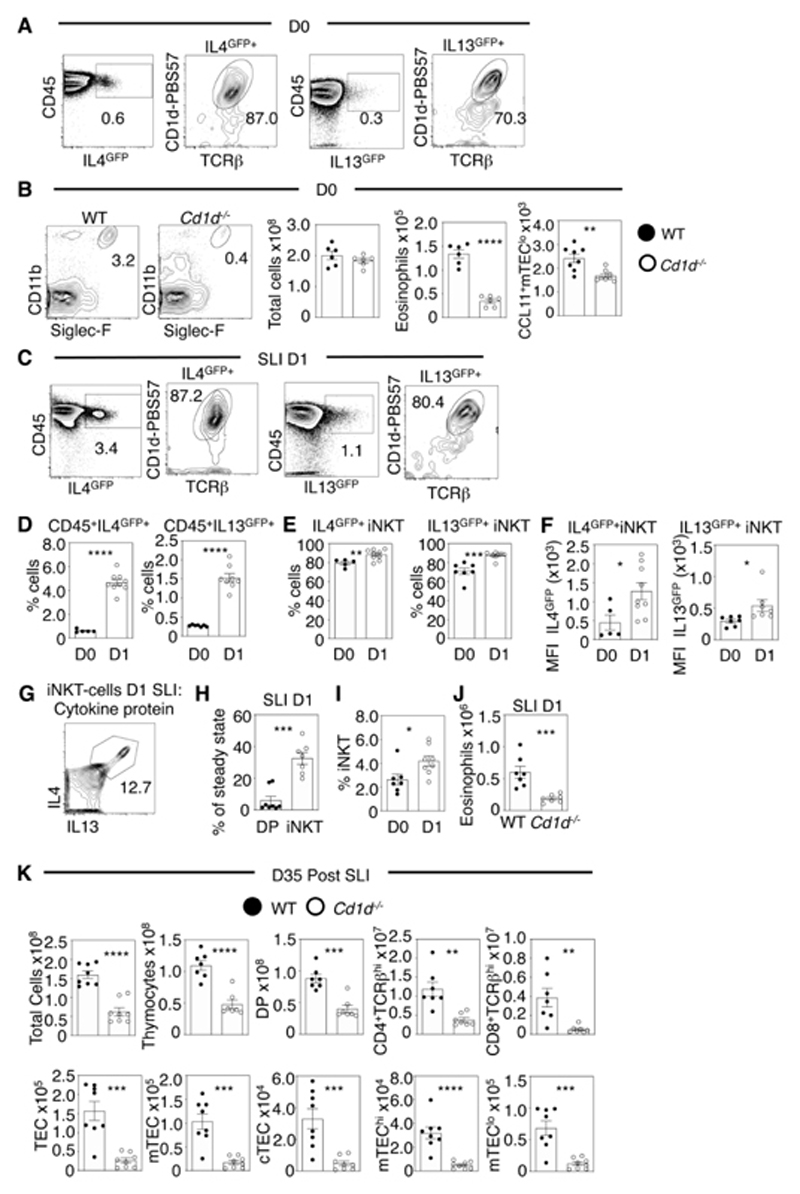
Type 2 Cytokine Production by iNKT-Cells Controls Thymus Regeneration. (A) Representative FACS plots showing IL4^GFP^ and IL13^GFP^ expression in total thymus cells and CD1d/PBS57 tetramer^+^ iNKT-cells at d0 (B). Representative FACs plots of eosinophils in WT and *Cd1d^-/-^* mice with quantitation of total cells, eosinophils and CCL11^+^ mTEC^lo^ at d0, n=6-8. Representative thymus FACS plots from IL4^GFP^ and IL13^GFP^ mice for GFP expression in total CD45^+^ cells and TCRβ^+^CD1d/PBS57 tetramer^+^ iNKT-cells at d1 (C). Proportions of CD45^+^ cells (D) and iNKT (E) expressing IL13^GFP^ and IL4^GFP^ at d0/d1 after SLI, n=5-9. (F) MFI levels of IL13^GFP^ and IL4^GFP^ in iNKT-cells at d0 and d1 post SLI, n=5-9. (G) Representative FACS plot of IL4, IL13 protein expression in thymus iNKT-cells at d1 post-SLI. (H) Relative proportions of iNKT-cell and CD4^+^8^+^ DP thymocytes in WT mice at d1 post-SLI compared to d0 steady state, n=7-8. (I) Proportion of thymic iNKT-cells at d0/d1 post SLI, n=7-8. (J) Thymic eosinophils at d1 after SLI in WT and *Cd1d^-/-^* mice, n=7. (K) SLI d35 *Cd1d^-/-^* mice analysis of T-cell development (top panel) and TEC (bottom panel), n=7-8. All data was from 2-3 independent experiments. All bars show mean ± SEM, * p<0.05, ** p<0.01, *** p<0.001, **** p<0.0001 from an unpaired students t-test.

The adult thymus supports the development of multiple αβT-cell types, including conventional αβT-cells, Foxp3^+^ Treg and CD1d-restricted iNKT-cells (28). Regarding the latter, the iNKT2 subset is a major intrathymic source of the cytokines IL4 and IL13 (23, 27, 29) that influence multiple thymus events including SP thymocyte emigration, DC maturation and SP CD8^+^Eomes^+^ cell production. Given the importance of IL4Rα signalling in thymus regeneration, we next examined the possible involvement of iNKT-cells in this process. Consistent with earlier studies, mCD1d-PBS57 tetramer positive iNKT-cells represented the majority of IL4^GFP+^ cells and IL13^GFP+^ cells in the thymus of unmanipulated IL13^GFP^ and IL4^GFP^ mice (Fig. 6A). Interestingly, thymus eosinophils were reduced in iNKT-deficient *Cd1d^-/-^* mice, as were CCL11^+^ mTEC^lo^ (Fig. 6B), suggesting iNKT-cells play a role in IL4Rα-mediated CCL11 production that controls thymus eosinophil availability.

To examine the role of IL4, IL13 and iNKT-cells in thymus regeneration, we performed SLI on IL4^GFP^ and IL13^GFP^ mice and analysed the thymus 1d post-SLI. Percentages of intrathymic IL4^GFP+^ and IL13^GFP+^ cells increased post-SLI (Fig. 6C, D), with both populations still dominated by iNKT-cells (Fig. 6E) that expressed higher GFP levels compared to untreated controls (Fig. 6F). In addition to IL4^GFP^/IL13^GFP^ reporter expression, we also detected IL4 and IL13 protein expression in iNKT-cells post-SLI (Fig 6G). Consistent with their relative radio-resistance compared to CD4^+^CD8^+^ thymocytes (Fig. 6H), percentages of iNKT-cells also increased 1d after damage (Fig. 6I). Thus, following SLI, iNKT-cells remain available in the thymus and represent an intrathymic source of IL4 and IL13 to trigger IL4R signalling. While the signals that control iNKT-cell cytokine production during thymus regeneration are not known, steady-state production of IL-4 by iNKT-cells is CD1d-dependent (30), raising the possibility that TCR signalling in iNKT-cells regulates their ability to make cytokines following damage.

To assess the role of iNKT-cells in thymus regeneration, we compared thymus recovery in WT and iNKT-cell deficient *Cd1d^-/-^* mice. At 1d post-SLI, thymus eosinophils were reduced in *Cd1d^-/-^* mice compared to SLI-treated WT mice (Fig. 6J). Moreover, at d35 post damage, *Cd1d^-/-^* mice showed significantly reduced thymus cellularity alongside reduced numbers of cTEC, mTEC and thymocytes (Fig. 6K). Such failures in thymus regeneration were seen in *Cd1d^-/-^* mice on both BALB/c and B6 backgrounds, arguing against a strain-specific mechanism of thymus recovery (Fig. S10A). Experiments with other gene knockout mice on both BALB/c and B6 backgrounds will extend our understanding of any background-specific effects on the mechanisms described here. Thus, iNKT-cell-deficient *Cd1d^-/-^* mice show defects in thymus regeneration that are accompanied by diminished intrathymic eosinophils. When we performed side-by-side comparative analysis of all available BALB/c strains in a single experiment (WT, *Cd1d^-/-^*, *Il4ra^-/-^*, ΔdblGATA), all showed failures in thymus generation, with the most significant defect in ΔdblGATA mice (Fig. S10B). Thus, alongside the involvement of iNKT-cells and IL4Rα, additional pathways may also link to the fundamental importance of eosinophils in thymus regeneration. While such pathways require further study, our current observations support a model in which iNKT-cells influence thymus regeneration through their IL4/IL13 production, which triggers IL4R signalling in thymic microenvironments and the recruitment of eosinophils to the thymus.

### Intrathymic IL5-Producing ILC2 Control Eosinophil-Mediated Thymus Regeneration

**Figure 7:**
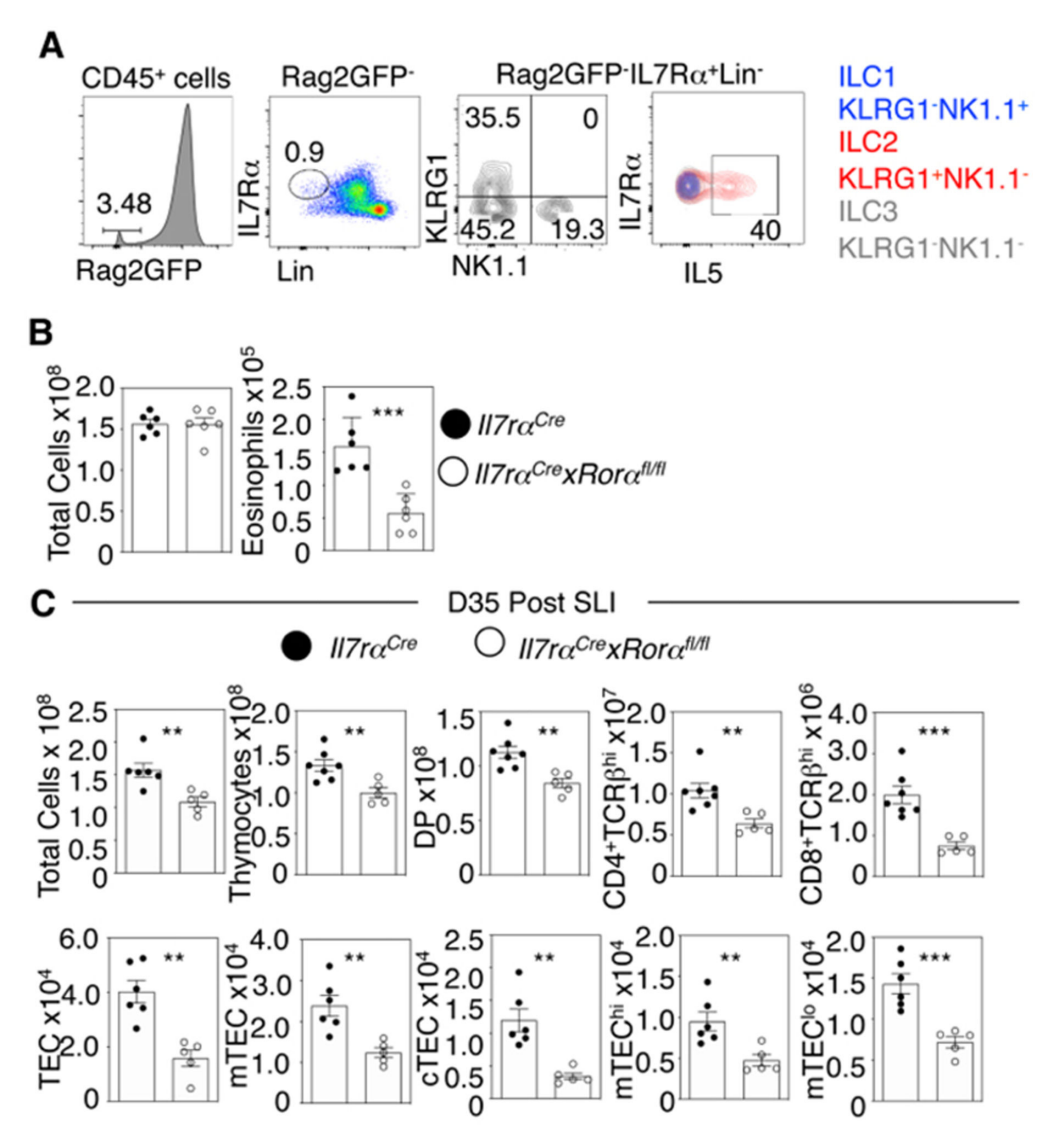
Impaired Thymus Regeneration in ILC2-Deficient Mice. (A) Red5xRag2GFP dual reporter mice were generated to identify intrathymic ILC subsets. GFP^-^ Lin^-^ (CD3, CD11b, CD11c, B220 and CD5) IL7Rα^+^ ILC were further subdivided into ILC1, 2 and 3 including KLRG1^+^NK1.1^-^L7Rα^+^ as indicated. Note the abundant and selective expression of IL5 by ILC. Data is representative of n=8 from three independent experiments. (B) *Il7ra^Cre^xRora^fl/fl^* ILC2-deficient mice compared to *Il7a^Cre^* controls at d0 for total thymic cellularity and number of thymic eosinophils, n=6 across at least two independent experiments. (C) Analysis of thymus recovery at d35 post SLI in *Il7ra^Cre^xRora^fl/fl^* ILC2-deficient mice compared to *Il7a^Cre^* controls, total thymus cellularity and thymocyte analysis (top panel) and TEC (bottom panel), n=5-7 across at least three experiments. All bars show mean ± SEM, ** p<0.01, *** p<0.001 from an unpaired students t-test.

As the findings above demonstrate the processes that control the increased availability of thymus eosinophils are necessary for thymus regeneration, we next examined intrathymic events that might help explain the requirement for eosinophils in this process. In many tissues, the cytokine interleukin-5 is a critical regulator of eosinophils, both in terms of their production, and their activation at sites of inflammation (31, 32). ILC2 are a known important source of IL5 and are present in multiple sites including thymus (33). To examine IL5 production by thymic ILC2, we crossed Rag2GFP with IL5^tdTomato^Red5 reporter mice. Here, flow cytometric gating to exclude the dominant population of immature GFP^+^ thymocytes enables the efficient detection of thymic ILC populations (Fig. 7A). Flow cytometric analysis of thymic cell suspensions from Rag2GFP/ IL5^tdTomato^Red5 dual reporter mice confirmed the presence of Lin^-^IL7Rα^+^KLRG1^+^NK1.1^-^ ILC2 in the thymus and identified them as an intrathymic source of IL5 (Fig. 7A). To examine whether ILC2 are linked to the requirement for eosinophils in thymus regeneration, we analysed *Il7ra* ^Cre^ *Rora* ^fl/fl^ ILC2-deficient mice (34). At steady state, while total thymus cellularity in ILC2-deficient mice and *Il7ra* ^Cre^ controls was comparable (Fig. 7B), we saw reduced numbers of thymic eosinophils in ILC2-deficient mice (Fig. 7B). Importantly, thymus regeneration in ILC2-deficient mice was significantly impaired following SLI (Fig. 7C).

**Figure 8:**
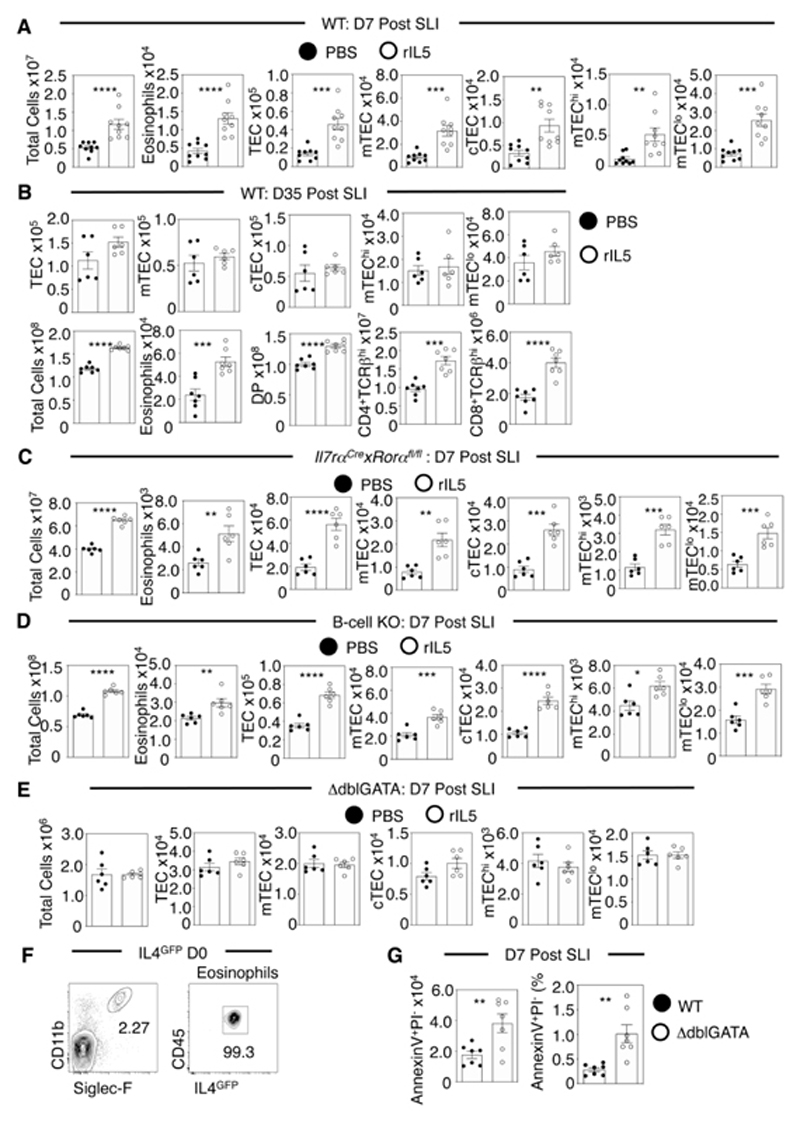
IL5 Administration Enhances Thymus Recovery in an Eosinophil Dependent Manner. (A) Analysis of thymus regeneration following PBS control/IL5 administration into WT BALB/c mice, completed at d7 post-SLI for total cells, eosinophils, and TEC, n=9 across three independent experiments. Similar analysis was performed at d35 post-SLI (B), with thymocyte analysis also completed at this timepoint, n=6-7 across 2 independent experiments. Analysis of thymus regeneration following PBS control or IL5 administration into *Il7ra^Cre^xRora^fl/fl^* mice (C), B-cell KO mice (D) and ΔdblGATA mice (E) at d7 post-SLI with analysis of total cells, eosinophils and TEC. n=6 for each strain across at least two independent experiments. (F) Representative FACs plots to show IL4^GFP^ expression by eosinophils in steady state IL4^GFP^ mice. (G) Annexin V and PI analysis of thymocytes at d7 post-SLI to assess dead and apoptotic cells in WT and ΔdblGATA mice, n=7 across two independent experiments. All bars show mean ± SEM, * p<0.05, ** p<0.01, *** p<0.001, **** p<0.0001 from an unpaired students t-test.

To see if this importance of ILC2 may link to their IL5 production, we first examined effects of IL5 administration in SLI-treated WT mice using the dosing regimen used for IL4. By d7 post-SLI, total thymus cellularity, thymus eosinophils and TEC numbers were significantly increased following recombinant IL5 treatment (Fig. 8A). Such effects were also evident at d35 post-SLI and accompanied by increased DP and CD4^+^ and CD8^+^ thymocytes (Fig. 8B). Thus, administration of IL5 to SLI-treated WT mice increases thymic eosinophils and improves thymus recovery following damage. Next, to determine whether the importance of ILC2 in thymus regeneration might specifically relate to their production of IL5, we performed the same IL5 dosing regime in SLI-treated ILC2-deficient mice. Importantly, this approach also enhanced thymus regeneration in ILC2-deficient mice, as indicated by increased thymic eosinophils, total thymic cellularity and TEC numbers compared to PBS controls (Fig. 8C). Collectively, such findings show that IL5 boosts thymus recovery after damage, and further indicate that an important role of ILC2 in this process maps to their production of IL5. The above findings indicate that ILC2 and their product IL5 are important regulators of thymus regeneration. To study the cellular target of administered IL5, we examined its effects on thymus recovery in SLI-treated mice deficient in either B-cells (35) or eosinophils (ΔdblGATA), both of which express IL5R in mice (36). Importantly, IL5 enhanced thymus regeneration in B-cell-deficient mice (Fig. 8D) but not eosinophil-deficient mice (Fig. 8E). Thus, the importance of the ILC2 product IL5 for thymus regeneration operates via a mechanism that is dependent upon eosinophils but independent of B-cells.

Collectively, the above findings identify the presence of a cellular network that regulates the recruitment of eosinophils to the thymus, that involves the actions of intrathymic innate cells including iNKT cells and ILC2 and their respective products IL4/IL13 and IL5. To examine further the mode of action of eosinophils during the regeneration process, we examined their expression of IL4, which has been linked to their requirement during liver regeneration. Flow cytometric analysis of eosinophils from IL4^GFP^ mice showed that thymic eosinophils were uniformly GFP^+^ (Fig. 8F), suggesting that once recruited to the thymus via the initial action of iNKT-cells, eosinophils may also trigger IL4Rα signalling to facilitate further eosinophil recruitment and/or operate directly on IL4Rα-expressing thymic stromal components to trigger regeneration. Finally, as eosinophils have been reported to play an important role in tissue regeneration via the clearance of apoptotic cells and cell debris (16) we performed SLI of ΔdblGATA and WT control mice and analysed the presence of apoptotic cells by flow cytometry 7d later (Fig. 8G). Interestingly, the number and percentages of apoptotic AnnexinV^+^PI^-^ cells was increased in ΔdblGATA mice compared to WT controls (Fig. 8G). This agrees with earlier observations that apoptotic cell clearance in the thymus involves eosinophils (37), and also might provide an explanation of the defective thymus regeneration seen in their absence.

## Discussion

The thymus represents an essential organ in both health and disease. Steady-state thymus function throughout life first establishes, then maintains, the peripheral T-cell pool that mounts effective anti-cancer and anti-pathogen immune responses. In disease, thymus function is essential for the re-establishment of the adaptive immune system, and thymus regeneration is a key process that ensures the recovery of thymus function (12, 13). Here, we show the thymus contains a multi-cellular network consisting of type 2 innate immune cells that restores T-cell production following damage. We show that in the acute response to SLI, radio-resistant iNKT release the type 2 cytokines IL4 and IL13. This then drives production of the chemokine CCL11 in mTEC to rapidly recruit eosinophils to the thymus via their expression of CCR3. In addition, as the repair response progresses, ILC2 contribute to the maintenance of eosinophils through their production of IL5. Moreover, this importance of ILC, and in particular their product IL5, is further emphasised by our finding that exogenous provision of IL5 accelerates thymic repair and T-cell replenishment.

The possible interactions that may occur between iNKT-cells and ILC2 that further explain their importance in thymus regeneration requires further investigation, and several scenarios exist. One possibility is that iNKT-cells and ILC operate at different stages in the same pathway. Here, iNKT-cells could operate upstream of ILC2 by releasing IL4/IL13 in response to CD1d ligation (30) which triggers CCL11 production in thymic stroma, and eosinophil recruitment to the thymus. Once inside the thymus, eosinophils are then influenced by intrathymic production of IL5 by ILC2. Alternatively, the involvement of iNKT-cells may extend beyond their initial role in eosinophil recruitment, and influence intrathymic eosinophils and/or ILC2 via their continued production of IL4/IL13 or other unknown factors. Moreover, in relation to how intrathymic ILC2 are controlled, it is interesting to note that ILC2 that reside in other tissues are regulated by alarmins such as IL25, TSLP and IL33 (26, 27, 38). Importantly, as thymic tuft cells are the exclusive intrathymic source of IL25 (26, 27), our finding that thymus regeneration occurs normally in *Pou2f3^-/-^* tuft cell deficient mice argues against an essential role for IL25 in the regulation of ILC2 following thymus damage. Further analysis of the potential importance of TLSP and IL33 will be valuable in assessing the molecular interplay that explains the involvement of ILC2 in thymus regeneration. While other studies have reported a role for ILC3 in thymus regeneration (13), the identification of a role for ILC2 extends our understanding of the importance of ILC in thymus biology. This demonstrates the functional relevance of innate immune components in the thymus, and emphasises the importance of cellular communication for thymus regeneration. Interestingly, the scenario of innate cell involvement in the regeneration of thymic microenvironments mirrors events that control the initial development and formation of embryonic TEC microenvironments. Here, a spectrum of innate cells that includes ILC3, invariant dendritic epidermal T-cells (DETC) and iNKT-cells have been demonstrated to play a role in the development thymus medulla areas (28). Thus, events that control thymus regeneration may recapitulate the processes that take place during homeostatic thymus development.

In steady state eosinophil-deficient ΔdblGATA mice, we found the thymus was able to support a normal programme of T-cell development. However, we noted a slight but significant reduction in splenic CD8^+^ αβT-cell numbers in these mice. Interestingly this was not detectable in *Il5^-/-^* mice that do contain eosinophils. Whether this difference relates to previous studies indicating the existence of IL5-dependent and IL-5 independent eosinophils in peripheral tissues (39), and a role for the former in controlling CD8^+^ αβT-cell numbers, is not clear. However, despite their non-essential role in steady state intrathymic T-cell development, we show an essential requirement for eosinophils in the recovery of thymus function following damage. This mirrors well their important repair functions in multiple tissues including muscle (16) and liver (17), indicating that non-lymphoid and lymphoid tissues share common regenerative mechanisms. How eosinophils regulate thymus generation is not fully understood. While identification of their essential role in this process provides an important platform for such work, further studies are needed to investigate this. Of particular importance is the kinetic of intrathymic eosinophil availability in relation to the kinetic of thymus recovery. We find that eosinophil numbers peak at 1 day post damage, and return to homeostatic levels by day 3 post SLI, suggesting that the effecter function of eosinophils may occur early after damage. It is also interesting to note that in the absence of eosinophils, thymus regeneration was still impaired at 98 day post-damage, suggesting that the absence of eosinophils in the early post-damage stages leads to a long-term inability of the thymus to recover. Such findings are compatible with several scenarios. First, eosinophils may be required to trigger an additional, as yet unknown, immune cell that directly controls TEC recovery. Second, and in line with their role in liver regeneration via effects on hepatocytes (17), eosinophils may act upon TEC or their progenitor populations that then regulate the long-term recovery of TEC microenvironments. Discriminating between these two possibilities will require a better understanding of the effecter functions of thymic eosinophils and innate thymic populations, as well as a clearer definition of the TEC progenitors that give rise to cTEC and mTEC compartments in the adult thymus._ Also, assessing the ability of transferred WT or genetically altered eosinophils to boost thymus regeneration is an attractive possibility. Perhaps relevant to the mechanism of action of eosinophils, we found thymic eosinophils were GFP^+^ in IL4^GFP^ reporter mice. Thus, alongside IL4 production by iNKT-cells, eosinophils may form part of a positive feedback loop where they stimulate CCL11 production via IL4Rα to regulate their recruitment to the thymus. In addition, while eosinophil involvement in muscle regeneration involves IL4/IL13 mediated effects on fibro/adipocyte progenitors (16), whether a similar mechanism occurs in thymus is not known. However, the same study also showed rapid clearance of cell debris was a requirement for effective tissue regeneration. Perhaps relevant to this, we found that the frequency of AnnexinV^+^PI^-^ early apoptotic cells was significantly increased in ΔdblGATA mice compared to WT. Thus, the requirement for eosinophils in thymus regeneration may link to their involvement in cell clearance, which supports previous suggestions this process may be an important component of thymus recovery (37, 40). If this is the case, how the transient intrathymic eosinophilia reported here then relates to mechanisms of apoptotic cell clearance is not known. Interestingly however, an important function of eosinophils may be their ability to produce and release DNA nets, known as eosinophil extracellular traps (EET). Such structures are known to persist in tissues following the disappearance of eosinophils (41), and may act as structures for the presentation multiple soluble factors that might aid in the clearance of apoptotic cells and/or cell debris.

Finally, how the mechanisms that operate in mouse thymus relate to thymus regeneration in man is unclear. Indeed, differences between the transcriptional profiles of human and mouse thymic cells have been reported (42). To our knowledge, it has not been examined whether human thymus regeneration is influenced by therapies such as benralizumab for eosinophil depletion or dupilumab for blockade of IL4/IL13 signalling, that target cellular and molecular components identified here. However, it is interesting that a child born to a mother receiving benralizumab had no noted immune defects, despite an absence of eosinophils during the first 6 months of life (43). While this is compatible with our observations in ΔdblGATA mice demonstrating a lack of requirement for eosinophils in steady-state thymus function, further work is needed to examine how such therapies may influence of human thymus recovery after damage.

In sum, our study identifies an intrathymic network of innate immune cells that collectively operates to guide the process of thymus regeneration following damage. Such findings provide a clearer understanding of a process that is essential to restore thymus function and T-cell production following insult. Our finding that administration of molecular components of this network, namely IL4 and IL5, are able to boost thymus regeneration further offers opportunities to manipulate these cells and their products for therapeutic benefit.

## Materials And Methods

### Study Design

The aim of this study was to gain understanding of the mechanisms that control the in vivo regeneration of thymus tissue following damage. Experimental approaches involved include multiparameter flow cytometry to quantitate defined cell populations, fetal thymus organ culture, qPCR for gene expression analysis, and cell culture and in vivo cell transfer for cell migration. Sample sizes used reflect previous and similar experiments as well as animal availability across the required strains. Numbers of replicates are provided in the figure legends, and there was no randomisation or blinding. All mice used were sex-matched (females).

### Mice

Mice (female, 8-10 weeks) were on either a BALB/c background: ΔdblGATA(18), *Cd1d^-/-^* (44), *Il4ra^−/−^* ((45), IL13^GFP^ (46), *Ccr3^-/-^* (47), *Il5^-/-^* (48), or a C57BL/6 background: IL4^GFP^ (4get) (49), IL5 tdTomato reporter (Red5) (50), Rag2pGFP transgenic (51), *Il7ra* ^Cre^ (52), *Rora* ^fl/fl^ (53), B-cell deficient μMT mice (35). For experiments that used mice on the BALB/c background, and in line with the policies of the University of Birmingham Biomedical Services Unit, commercially available or donated strains imported into Birmingham were intercrossed with BALB/cnNCrl mice available in the UK from Charles River. In all cases, BALB/cnNCrl mice were used as WT controls. For these reasons, and in line with other studies examining immune regulation and/or thymus regeneration using BALB/c mice (13, 54), we refer to these models as BALB/c throughout the study. Husbandry, housing, and experimental methods involving mice were performed at the Biomedical Services Unit at the University of Birmingham, in accordance with local ethical review panel and national Home Office regulations.

### Flow Cytometry and Cell Sorting

For eosinophil analysis, cells were obtained from thymus and spleen tissues following enzymatic digestion using 2.5mg/ml Collagenase D (Roche) and 100mg/ml DNase-I (Roche). Lung samples were prepared using 12.5μg/ml Liberase TM (Roche) and 100mg/ml DNase-I (Roche). Small intestine samples were obtained and cut into 0.5cm sections and washed with Hank’s Balanced Salt Solution (HBSS, Gibco) with 2% foetal bovine serum. Tissue was incubated with HBSS 2mM EDTA at 37°C to remove epithelial cells and then digested in complete RPMI (Gibco) with 1mg/ml Collagenase VIII (Sigma) at 37°C. For thymocyte, NKT and ILC analysis, thymi were mechanically disaggregated prior to surface staining. Reagents used were: anti-CD45 (30-F11; eBioscience), anti-TCRβ (H57-597; eBioscience), anti-CD11b (M1/70; Biolegend), anti-Siglec-F (ES22-10D8; Miltenyi Biotech), anti-CD4 (RM4-5; Biolegend), anti-CD8α (53-6.7; Biolegend), anti-TER119 (TER-119; Biolegend), anti-CCR3 (J073E5; Biolegend), anti-CD69 (H1.2F3; eBioscience), anti-CD62L (MEL-14; eBioscience), PBS57/mCD1d tetramer (National Institutes of Health Tetramer Core Facility), anti-IL7Rα (A7P34; eBioscience), anti-KLRG1 (2F1/KLRG1; Biolegend). Lineage panel used was anti-CD3ε (145-2C11; eBioscience), anti-CD5 (53-7.3; eBioscience), anti-CD11b (ICRF44; Invitrogen), anti-CD11c (N418; Invitrogen), anti-B220 (RA3-6B2; Invitrogen), anti-NK1.1 (PK136; Invitrogen). For TEC, mesenchyme and endothelial analysis (55), thymus tissues were digested with 2.5mg/ml collagenase dispase (Roche) and 100mg/ml DNAse -I (Roche) before being stained with the following antibodies/reagents: anti-EpCAM1 (G8.8; eBioscience), UEA1 (Vector Labs), Streptavidin (eBioscience), anti-Ly51(BP-1; BD Pharmingen), anti-MHC-II (M5/114.15.2; eBioscience), anti-CD80 (16-10A1; Biolegend), gp38(8.1.1 eBioscience), CD31(ebio390/390 eBioscience). For PCR analysis, adult TEC subsets were sorted using a BD Aria sorter as bulk mTEC (CD45^-^EpCAM1^+^UEA1^+^Ly51^-^), mTEC^hi^ (CD45^-^EpCAM1^+^UEA1^+^Ly51^-^MHCII^hi^CD80^hi^), mTEC^lo^ (CD45^-^EpCAM1^+^UEA1^+^Ly51^-^ MHCII^lo^CD80^lo^), and cTEC (CD45^-^EpCAM1^+^UEA1^-^Ly51^+^). PCR analysis of IL4/IL13 stimulated FTOC was performed after disaggregation with 0.25% trypsin (56).Where indicated, intracellular staining was performed using the Foxp3/transcription factor staining buffer set (eBioscience), according to manufacturer’s instructions. Intracellular staining was performed using purified Goat IgG anti-CCL11 (AF-420-NA; R&D Systems), and secondary antibody chicken anti-goat Alexa Fluor 488 IgG (AF21467, Invitrogen) and DCLK1 (DCLKL1, AbCam) followed by donkey anti-rabbit 647 (A21443, Invitrogen). All flow cytometric data was analysed on a BD Fortessa, using FlowJo version 8.7.3.

### Sub-Lethal Irradiation (SLI)

Mice on a BALB/c background were subjected to sub-lethal total body irradiation with a dose of 1x425rad (4.25Gy) (CIS BIO International, Cedex, France); C57BL/6 background were given 1x500 rad (5.0 Gy). Mice were given Baytril for one week prior to sub-lethal irradiation as well as one week after. Mice were then harvested at indicated time points to assess thymus recovery.

### Eosinophil Cultures and Cell Transfer

Eosinophils were generated *in vitro* as described (57). In brief, bone marrow cells were obtained from femurs and tibiae of WT BALB/c mice by flushing with RPMI containing 10% FCS (Sigma). Red blood cells were lysed and 1x10^6^ cells were cultured in Dulbecco’s Modified Eagle’s Medium with 10% FCS, supplemented with 100ng/ml stem cell factor (PeproTech) and 100ng/ml FLT3Ligand (PeproTech) from culture days 0-4. Cultures were changed on day 4 to medium containing 10ng/ml recombinant murine IL5 (R&D Systems) and fed with IL5 until day 10-14. Cells were then collected (typically containing more than 90% eosinophils), counted, and labelled with 5μM Cell-Trace Violet (CTV, Invitrogen) at 37°C. In some experiments, eosinophils were treated with 10mM of the CCR3 antagonist (SB328437, R&D Systems) for 1hour at 37°C, prior to CTV labelling and i.v. transfer. 8-10x10^6^ cells were i.v. injected into mice at either steady state or 4 hours after irradiation. Mice were harvested 24hours after irradiation, with non-irradiated controls taken at the same time.

### Recombinant IL5 / Recombinant IL4 Injections

WT BALB/c mice were i.p. injected with either PBS or 40ng of recombinant IL5/recombinant IL4 (PeproTech) on days -3, -2, -1 and +1, with the time point of 0 set as the time of SLI.

### Cell stimulations

For intracellular cytokine staining of IL4 and IL13, suspensions of D0 and D1 SLI thymocytes were cultured for 3 hours with Brefeldin A (10 μg/ml) (Sigma) alone. An additional positive control was run alongside whereby thymocytes from D0 and D1 SLI mice were stimulated *in vitro* with Ionomycin (1.5 μM) and phorbol myristate acetate (PMA) (50 μg/ml), for the positive controls, with the addition of Brefeldin A (10 μg/ml) (all Sigma–Aldrich) for 3h. All samples were then antibody stained for IL4 and IL13.

### Annexin V/PI staining

To determine dead or apoptotic cells following SLI exposure, Annexin V and PI were used to stain D0 and D1 post SLI cells in WT and ΔdblGATA mice. The kit was purchased from Invitrogen and used as per suggested protocol.

### Fetal Thymus Organ Culture

E15 thymic lobes obtained from WT BALB/c embryos were organ cultured for 5-7 days in 1.35mM 2-deoxyguanosine (2-dGuo, Sigma), as described (58). Lobes were then cultured for an additional 4 days in the presence or absence of 100μg/ml recombinant IL4 (BioLegend) and/or IL13 (PeproTech). Lobes were digested with 0.25% trypsin/0.02% EDTA (Sigma-Aldrich) and depleted of any remaining CD45^+^ with Dynabeads (Dynal, Thermofisher), and stromal cells were snap frozen for quantitative PCR (qPCR).

### Quantitative Polymerase Chain Reaction

High-purity complementary DNA (cDNA) was obtained from mRNA labelled with oligo(dT) microbeads using the μMacs One-step cDNA Kit, according to the manufacturer’s instructions (Miltenyi Biotec). Real-time PCR was performed as described previously (59), with SensiMix SYBR No ROX Kit (Meridian Bioscience) with primers specific for *Actb* (Qiagen) and *Ccl11* (Sigma-Merck) on the Corbett Rotor Gene-3000 PCR machine (Qiagen). Fold levels shown in histograms represent the mean (±SEM) of replicate reactions and data shown are representative of at least three independently sorted sample sets. *Ccl11* primer sequences used: forward 5’- AGAGCTCCACAGCGCTTCTA -3’ reverse 5’-GGAAGTTGGGATGGAGCCTGG -3’. *Ccl24* primer sequences used: forward 5’-ATTCTGTGACCATCCCCTCAT -3’ reverse 5’-TGTATGTGCCTCTGAACCCAC-3’. *Actb* (β-actin) was measured using the QuantiTect Mm *Actb* 1SG Primer Assay (Qiagen, QT00095242).

### Statistical Analysis

Prism 9 (GraphPad Software) was used to perform all statistical analyses. Unpaired students t-test was used for comparisons between two data sets, and graphs were annotated to indicate significance. For comparison of three or more groups a one-way ANOVA was performed with Bonferroni post-test analysis. All statistical significance is listed as follows: *, P < 0.05; **, P < 0.01; ***, p < 0.001; and ****, P < 0.0001. Nonsignificant differences were not specified. In all figures, bar charts and error bars represent the means ± SEM.

## Supplementary Material

Fig. S1

Table S1

## Data Availability

All data needed to support the conclusions of the paper are available in the paper or the Supplementary Materials.

## One Sentence Summary

Eosinophils are a critical component of an innate immune network that drives recovery of thymus function following damage.
