# Supplementary material for: Eosinophils Are An Essential Element Of A Type 2 Immune Axis That Controls Thymus Regeneration
Source: Sci Immunol. Author manuscript; Available in PMC 2022 Apr 4. (PMC7612579; doi:10.1126/sciimmunol.abn3286)
Supplement: Fig. S1 [file EMS144040-supplement-Fig__S1.pdf]

Science Immunology
AAAS

Supplementary Materials for

Eosinophils are an essential element of a type 2 immune axis that controls thymus regeneration

Emilie J. Cosway *et al.*

Corresponding author: Graham Anderson, g.anderson@bham.ac.uk

Sci. Immunol. **7**, eabn3286 (2022)
DOI: 10.1126/sciimmunol.abn3286

The PDF file includes:

Figs. S1 to S10

Other Supplementary Material for this manuscript includes the following:

Table S1
MDAR Reproducibility Checklist

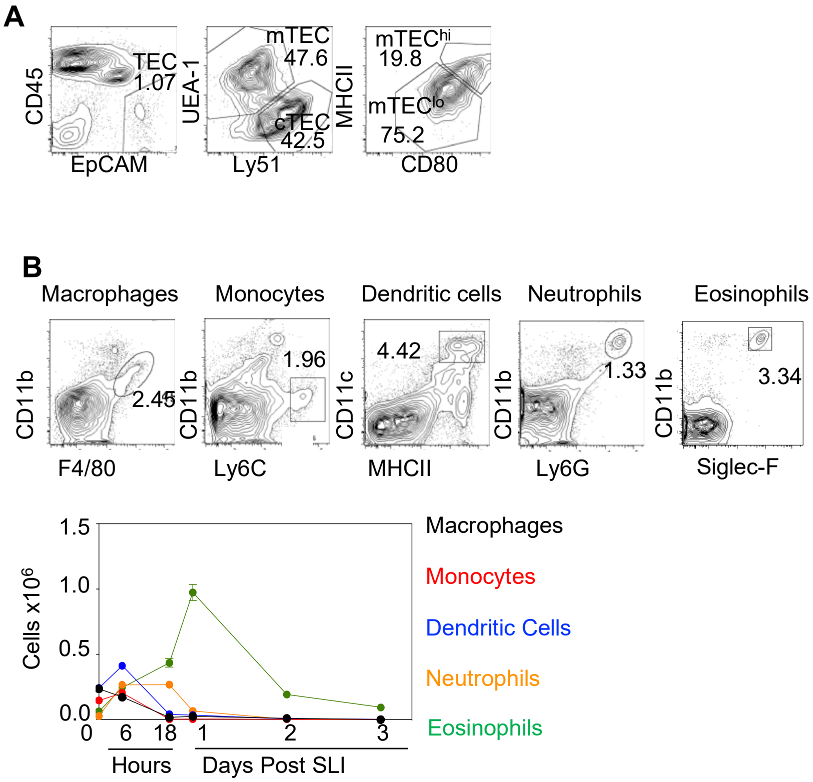

Figure S1. Time course of innate cells in thymus after SLI.

(A) Representative FACs plots for identification of TEC and TEC subsets; TEC ($CD45^-$ $EpCAM^+$), mTEC ($UEA\text{-}1^+Ly51^-$), cTEC ($UEA\text{-}1^-Ly51^+$), $mTEC^{hi}$ ($UEA\text{-}1^+Ly51^-MHCII^+CD80^+$), $mTEC^{lo}$ ($UEA\text{-}1^+Ly51^-MHCII^-CD80^-$). (B) Cell types were identified as follows; $CD45^+Siglec\text{-}F^-Ly6G^-CD11b^+F4/80^+$ (macrophages), $CD45^+Siglec\text{-}F^-CD11b^+Ly6C^+$ (monocytes), $CD45^+Siglec\text{-}F^-CD11c^+MHCII^+$ (dendritic cells), $CD45^+Siglec\text{-}F^-CD11b^+Ly6G^+$ (neutrophils) and $CD4^-CD8^-TCRb^-TER119^-CD11b^+Siglec\text{-}F^+$ (eosinophils). Data is representative of a minimum n=8 across at least two independent experiments.

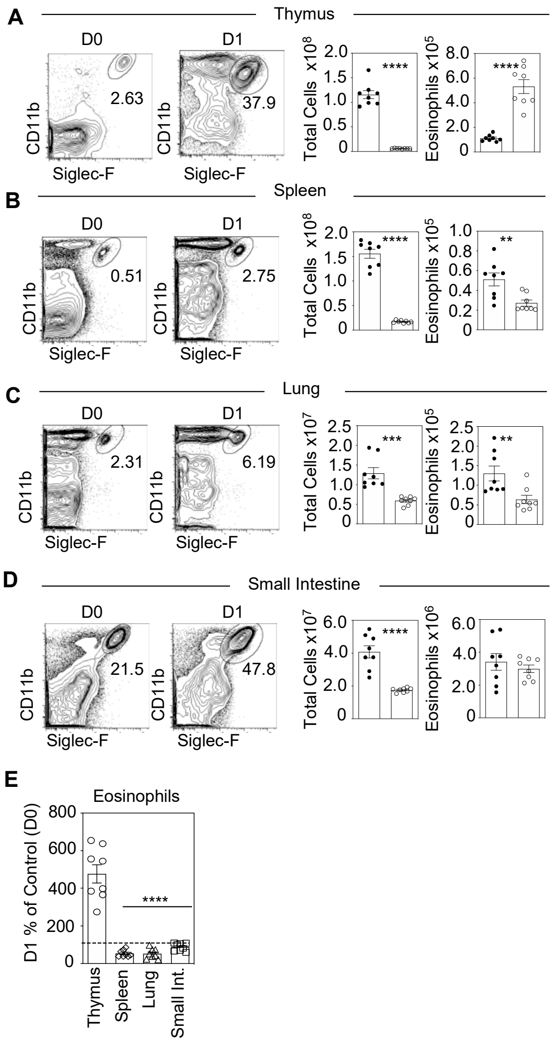

Figure S2. Quantitation of eosinophils in multiple tissues following SLI.

Representative FACS plots and absolute numbers of total organ cellularity and eosinophils at steady state (D0) and D1 after SLI in thymus (A), spleen (B), lung (C) and small intestine (D). (E) Summary of eosinophil frequency at D1 post SLI, where dashed line at 100% represents frequencies in untreated mice. Analysis is from two experiments where n=8. All bars show mean ± SEM, ** $p<0.01$, *** $p<0.001$, **** $p<0.0001$ from an unpaired students t-test.

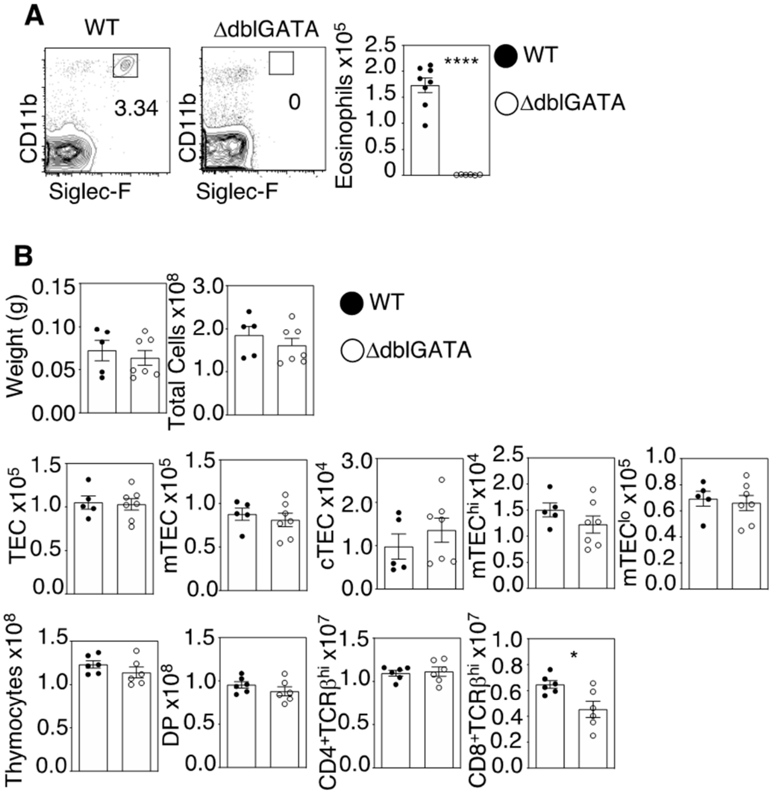

Figure S3. Thymus analysis of steady state DdblGATA and WT adult thymus.

(A) Analysis of thymus eosinophils in WT Balb/c and DdblGATA mice at D0. Eosinophils were identified as CD4^{-}CD8^{-}TCRβ^{-}TER119^{-}CD11β^{+}Siglec-F^{+} cells, n=6-8 across two independent experiments. (B) Analysis of TEC populations (top panel) (n=5/7) or thymocytes (bottom panel) (n=6) in steady state D0 WT and ΔdblGATA mice from at least two experiments. All bars show mean ± SEM, * $p<0.05$, **** $p<0.0001$ from an unpaired students t-test.

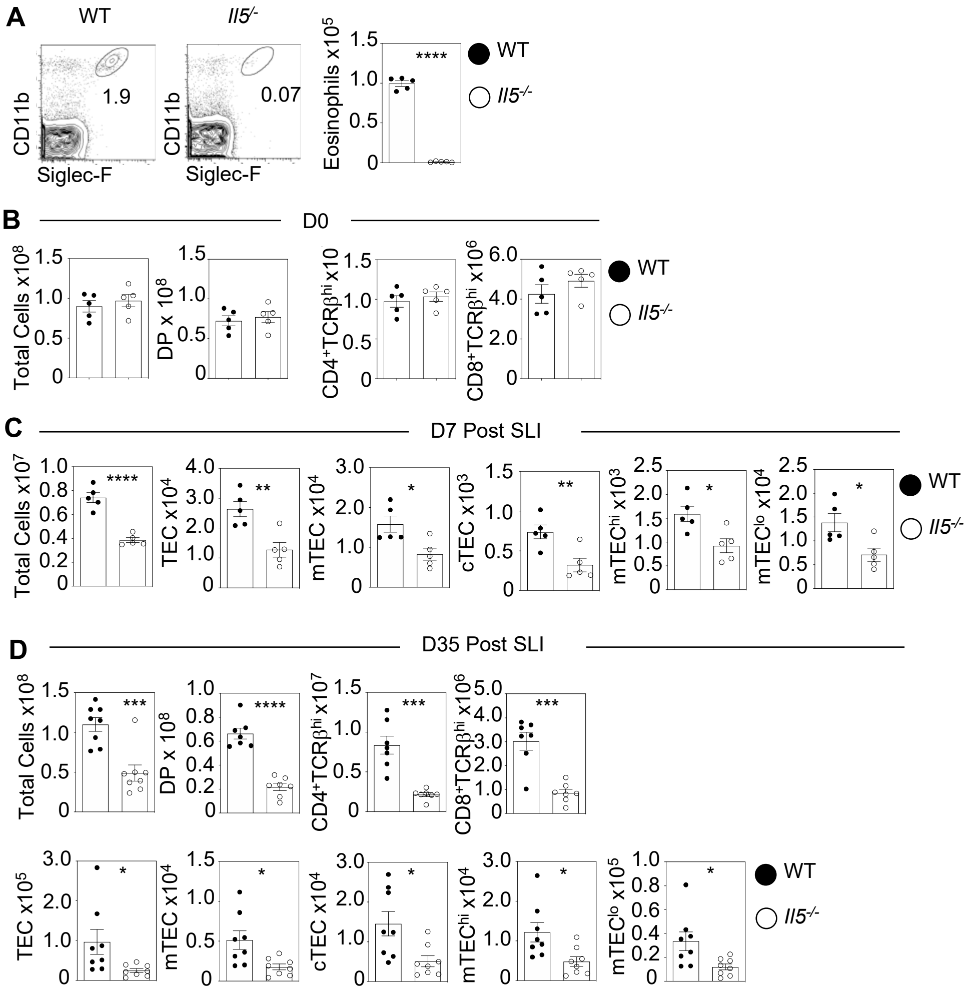

Figure S4. Failure of thymus regeneration in *Il5* mice.

(A) Quantitation of thymus eosinophils in steady state *Il5*$^{ï/ï}$ mice, n=5 across two independent experiments. (B) Steady state analysis of WT and *Il5*$^{ï/ï}$ mice in thymus for thymocyte development n=5, across two independent experiments. (C) Recovery of thymus D7 post SLI in WT and *Il5*$^{ï/ï}$ mice, for TEC analysis n=5, across two independent experiments. (D) Recovery of thymus D35 post SLI for TEC and T-cell development, n=7-8 across two independent experiments. All bars show mean ± SEM, * $p<0.05$, ** $p<0.01$, *** $p<0.001$, **** $p<0.0001$ from an unpaired students t-test.

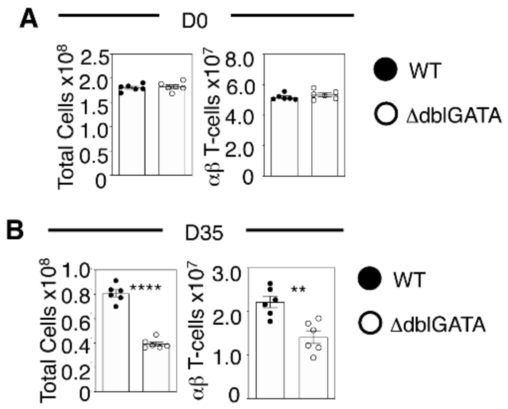

Figure S5. Impaired recovery of the peripheral T-cell pool in SLI-treated ΔdblGATA mice. Absolute number analysis of T-cell populations in the spleen of WT and ΔdblGATA mice following SLI, analysis at D0 (A), D35 (B) including total splenic cellularity and αβT-cell analysis. Data is from two independent experiment, n=6. All bars show mean ± SEM, ** $p<0.01$, **** $p<0.0001$ from an unpaired students t-test.

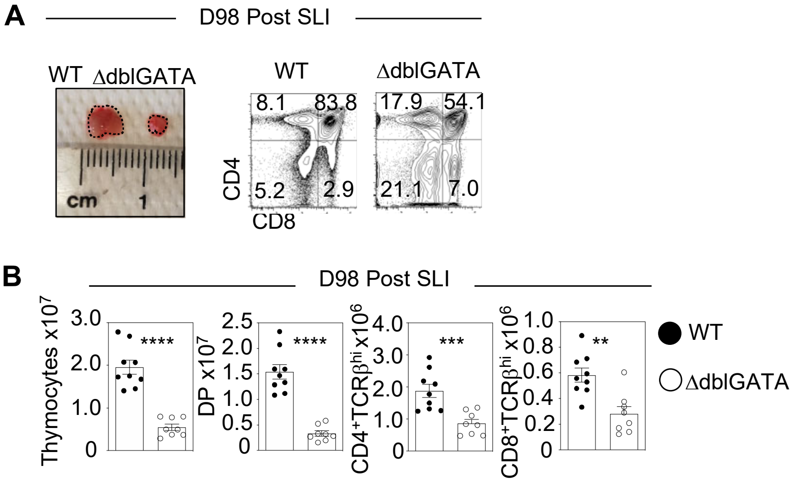

Figure S6. Eosinophils are essential for long term thymus recovery after SLI induced damage. (A) WT mice were subjected to SLI and thymus was harvested at D98 post SLI. Images show gross thymus size and representative FACS plots of T-cell development at D98 post SLI. (B) Analysis of absolute numbers of thymocytes at D98 post SLI in WT and ΔdblGATA mice. Data was generated across at least two independent experiments where n=8-9. All bars show mean ± SEM, ** $p<0.01$, *** $p<0.001$, **** $p<0.0001$ from an unpaired students t-test.

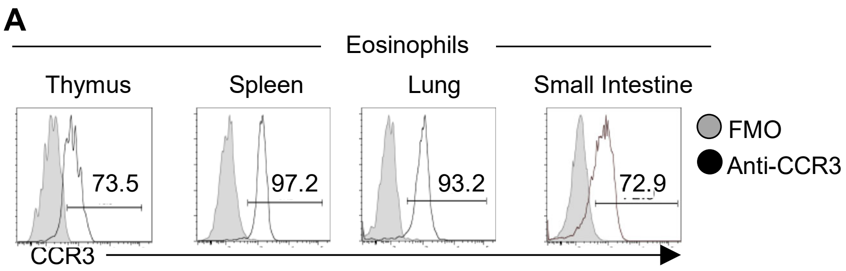

Figure S7. CCR3 expression by eosinophils in tissues.

(A) Representative FACS plots to show the expression of CCR3 on eosinophils against an FMO (grey filled), identified as $CD4^{-}CD8^{-}TCR\beta^{-}TER119^{-}CD11b^{+}$Siglec-$F^{+}$. Data is representative of n=6 across two independent experiments.

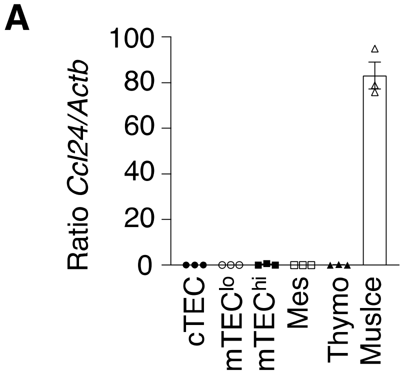

Figure S8. Absence of CCL24 expression in TEC, thymic mesenchyme and thymocytes.

(A) qPCR analysis of *Ccl24* mRNA expression in WT adult cTEC, mTEC^{lo}, mTEC^{hi}, thymic mesenchyme (Mes) and thymocytes (thymo) and muscle. Three independent biologically sorted samples were used for each tissue, ran in replicates with the mean +/-SEM plotted.

A

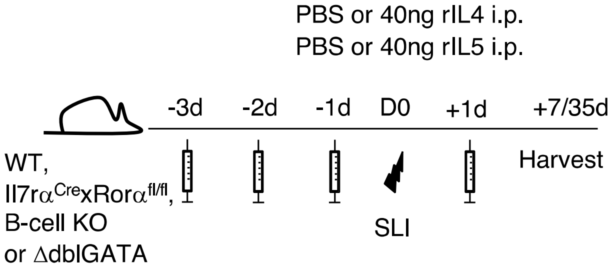

Figure S9. Cytokine dosing regime for thymus regeneration.

(A) Mice were subjected to a time-course of injections, three prior to SLI exposure at D0 and then one the following day. Mice were either injected with PBS as a control or 40ng of recombinant IL4 (rIL4) or recombinant IL5 (rIL5).

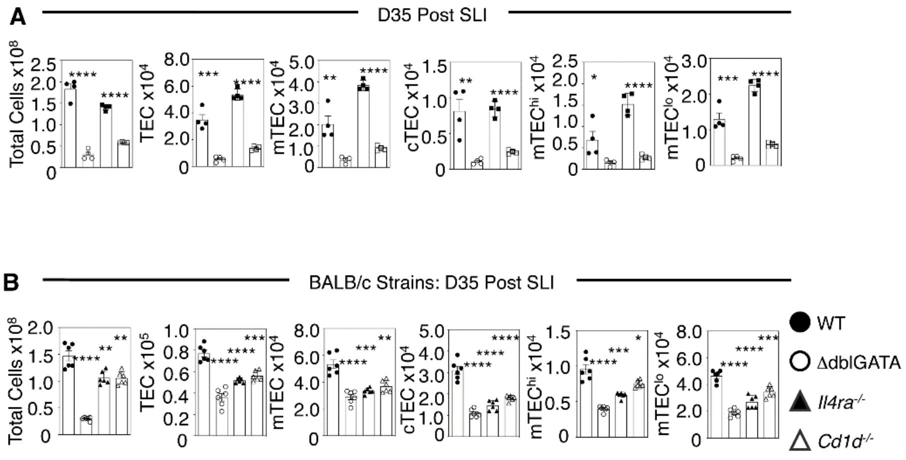

Figure S10. Comparison of recovery D35 after damage between different mouse strains on different backgrounds.

(A) D35 post SLI TEC analysis comparing the recovery of thymus between WT and *Cd1d*$^{-/-}$ mice on either a B6 or Balb/c Background. Statistical analysis performed compares WT to *Cd1d*$^{-/-}$ mice on the same background, data is obtained from 2 independent experiments where n=4. (B) TEC analysis of thymus recovery in gene knockout and WT mice on a BALB/c background, D35 post SLI exposure with significance relating to knockout mice compared to WT counterparts. Data is representative of n=6 across 2 independent experiments. All bars show mean ± SEM, * $p<0.05$, ** $p<0.01$ *** $p<0.001$, **** $p<0.0001$ from an unpaired students t-test.
